# An unusual presentation of fucosidosis in a Chinese boy: a case report and literature review (childhood fucosidosis)

**DOI:** 10.1186/s12887-022-03414-y

**Published:** 2022-07-11

**Authors:** Shao-Jia Mao, Jia Zhao, Zheng Shen, Chao-Chun Zou

**Affiliations:** 1 Department of Endocrinology, the Children’s Hospital of Zhejiang University School of Medicine, No 3333 Binsheng Road, Hangzhou, 310051 Zhejiang Province China; 2 Department of Pediatrics, the People’s Hospital of Zhuji, Shaoxing, Zhejiang Province China

**Keywords:** Fucosidosis, Lysosomal storage disease, α-L-fucosidase, All tissue systems, FUCA1

## Abstract

**Background:**

Fucosidosis is one of the rare autosomal recessive lysosomal storage diseases (LSDs) attributed to FUCA1 variants causing the deficiency of α-L-fucosidase in vivo. Α-L-fucosidase deficiency will cause excessive accumulation of fucosylated glycoproteins and glycolipids, which eventually leads to dysfunction in all tissue systems and presents with multiple symptoms. Fucosidosis is a rare disease which is approximately 120 cases have been reported worldwide (Wang, L. et al., J Int Med Res 48, 1-6, 2020). The number of reported cases in China is no more than 10 (Zhang, X. et al., J Int Med Res 49:3000605211005975, 2021).

**Case presentation:**

The patient was an 8-year-old Chinese boy who presented with postnatal motor retardation, intellectual disability, short stature, language development retardation, coarse facial features, hepatomegaly, and diffuse angiokeratoma of both palms. His genetic testing showed the presence of a homozygous pathogenic variant (c.671delC) in the FUCA1 gene. In addition, the enzymatic activity of α-L-fucosidase was low. Ultimately, the patient was diagnosed with fucosidosis.

**Conclusions:**

Fucosidosis is a rare lysosomal storage disease because of FUCA1 variants that cause the deficiency of α-L-fucosidase in vivo. An explicit diagnosis requires a combination of clinical manifestations, imaging examination, genetic testing and enzyme activity analysis. Early diagnosis plays an important role in fucosidosis.

## Background

Fucosidosis (OMIM 230,000) is one of the autosomal recessive lysosomal storage diseases (LSDs) caused by variants of FUCA1 (NG_013346) on chromosome 1p36.11 [1]. The FUCA1 gene encodes α-L-fucosidase (EC 3.2.1.51). Fucosidosis is a rare disease which is about 120 cases have been reported worldwide. A-L-fucosidase is a lysosomal enzyme that degrades fucosylated glycoproteins and glycolipids in vivo. The deficiency of α-L-fucosidase leads to the excessive accumulation of fucosylated glycoproteins and glycolipids in multiple tissues and organs, such as brain, bone, skin, and liver, causing multiple clinical symptoms. Most patients have intellectual disability, dyskinesia, coarse facial features, recurrent respiratory infections, growth retardation, and dysostosis multiplex [2]. These symptoms are similar to those of other LSDs, such as mucopolysaccharidosis (MPS) and mucolipidosis (ML). In addition, some patients have diffuse telangiectasia or angiokeratomas which has been observed more often in older patients [1, 3, 4]. Moreover, other symptoms, such as hepatosplenomegaly, epilepsy, and inguinal hernia, are often manifested in patients with fucosidosis. According to onset age and clinical features, fucosidosis is divided into two types. Type I usually begins early (before the age of one year) and progresses relatively quickly. In addition, the deterioration of the nervous system is faster than that of type II. Typical patients of Type I tend to die between the ages of 5 to 10. Type II often begins later than 2 years old, progresses more slowly, and usually allows patients to survive until adulthood [1, 5]. However, because fucosidosis is extremely rare and has nonspecific clinical manifestations, the diagnosis of these patients is often delayed. Herein, we reported an 8-year-old Chinese boy with fucosidosis who was previously misdiagnosed with MPS.

## Case presentation

An 8-year-old boy visited our unit for "motor and intellectual retardation". He was G1P1 (first pregnancy & first delivery) of non-consanguineous healthy parents and was delivered naturally at term with a birth weight of 3.25 kg (-0.39 SD). The movement retardation was noted after birth. He was not able to walk until the age of 18 months. Up to now, he continued to walk unsteadily and unsymmetrically. Moreover, he could only unconsciously utter the word "mother", and his intellectual disability prevented conscious communication. The growth retardation also was noted that the growth rate was only 2 cm per year in recent years. Since the age of 3 years, there have been dense red rashes on the palms of both hands. The rashes spread from hand to trunk. The patient used to suffer from recurrent respiratory infections. He had a right inguinal hernia at 5 years old and then underwent high ligation of the hernia sac under the laparoscope in the same year. No similar genetic family history was reported.

**Fig. 1:**
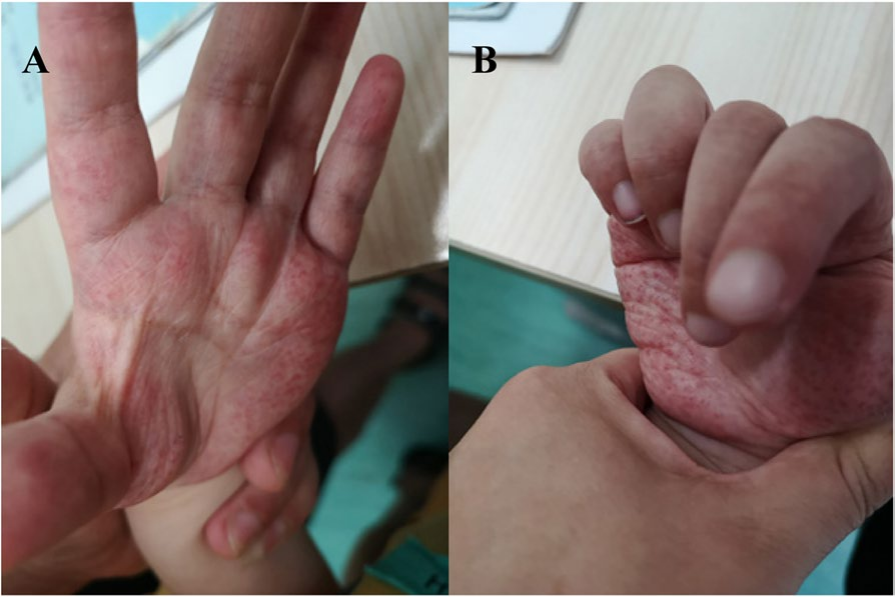
Clinical manifestations of the patient. **A** Palms with a dense red rash; (**B**) Fingers that couldn't be fully extended

Physical examination showed a height of 116.5 cm (-2.48 SD) and a weight of 25 kg (-0.60 SD). The ability to communicate was missing. He had dull eyes, slightly coarse facial features, black hair, and thick eyebrow. The liver was relatively enlarged and about 3 cm under the ribs. The spleen was not palped under the ribs. Muscular hypotonia was noted. Each finger couldn’t be fully extended (Fig. 1A). Numerous red rashes were seen on both palms (Fig. 1B). Cardiopulmonary auscultation showed no abnormality. The muscle strength of the limbs was normal. Eye manifestations were undetected. No other obvious abnormality was found.

**Fig. 2:**
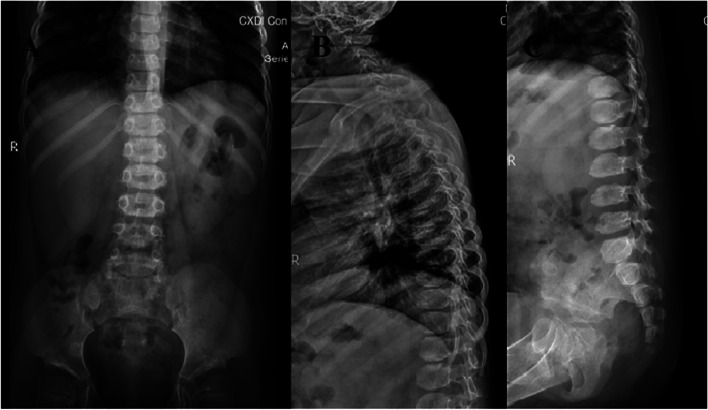
Anterior lateral radiograph of the spine of the patient. **A** concave proximal ends of the ribs, the irregular shape of the centrum and bilateral shallow acetabulum with irregular margin; (**B**) and (**C**) anterior beaking of the lower thoracic and lumbar vertebrae

The hepatosplenic ultrasound showed hepatomegaly with the length of 4.5 cm under the ribs. Echocardiography indicated mild tricuspid regurgitation without valve thickening or myocardial hypertrophy. X-ray of the spine revealed the presence of physiological curvature of the spine, tapering of the anterior vertebral body with irregular morphology, and local bone hyperplasia of the vertebral body. The proximal end of the ribs was concave. Each acetabulum was shallow, and its margin was irregular (Fig. 2A-C). In the brain Magnetic Resonance Imaging(MRI), the globus pallidus showed symmetrical hyperintensity on T1-weighted images and symmetrical hypointensity on T2-weighted images with a linear high-intensity zone in the middle. The volume of cerebral white matter was decreased slightly. Liver and kidney function, blood gas, electrolyte, blood glucose, thyroid function, and the genetic metabolic indexes of peripheral blood were all normal.

**Fig. 3:**
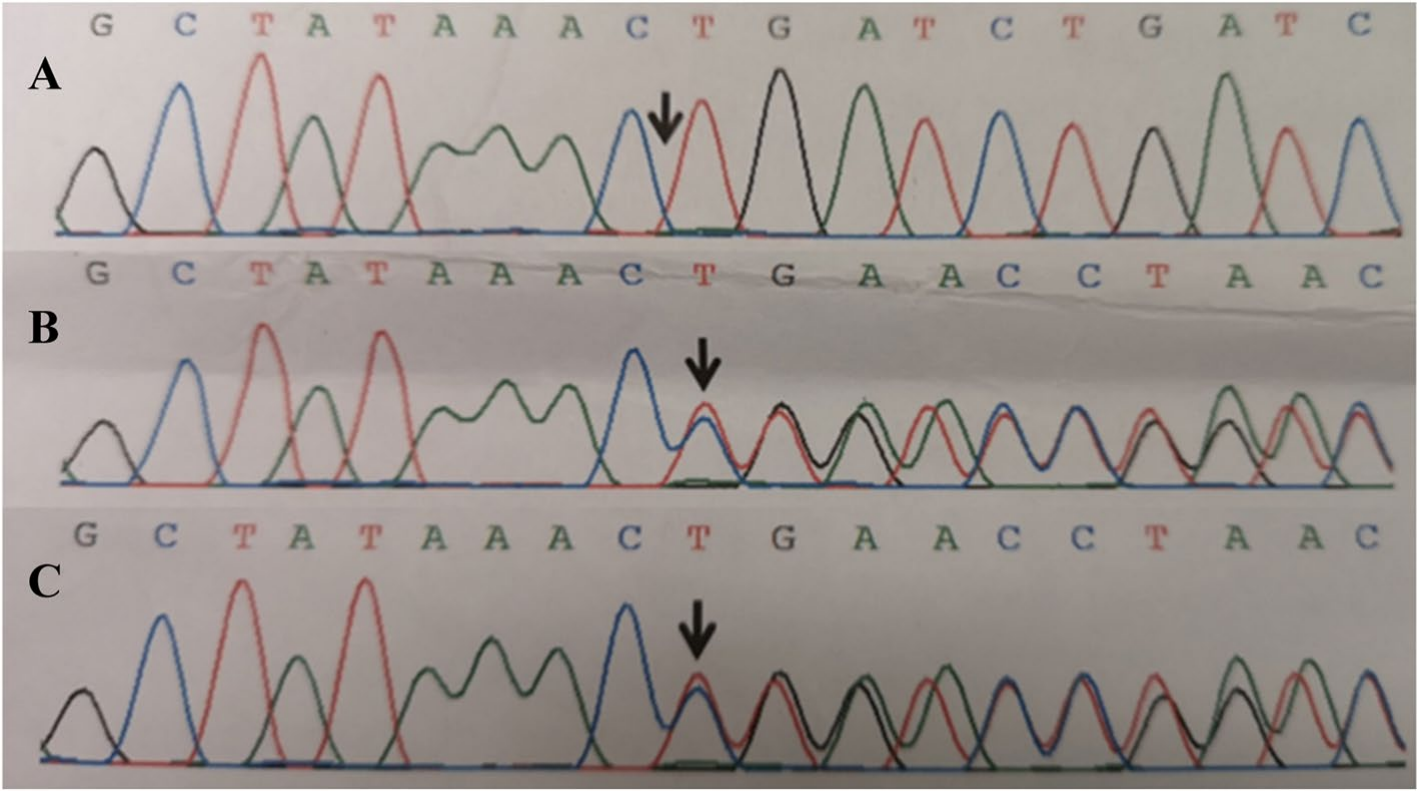
Sanger sequencing chromatograms of FUCA1 variants in the patient's family. **A** Homozygous c.671delC variant in the FUCA1 gene of the patient; (**B**) Heterozygous c.671delC variant in the FUCA1 gene of his father; (**C**) Heterozygous c.671delC variant in the FUCA1 gene of his mother

MPS was initially suspected. Urinary glycosaminoglycans (GAGs) screen for MPS was performed by dimethylmethylene blue dye binding assay with the boundary result. Hence, other genetic diseases (e.g., myxolipomatosis or fucosidosis) were considered. Whole exon sequencing performed for the boy and his parents showed a presence of a homozygous pathogenic variant (c.671delC in exon 4) of FUCA1 gene, inherited from his parents (Fig. 3A-C). The c.671delC is a de novo and frameshift pathogenic variant that causes a premature stop codon (p.Pro224LeufsTer3). This variant was not reported previously in GnomAD data and was regarded as pathogenic according to the ACMG criterion. In order to make sure, the α-L-fucosidase activity analysis was done that the level of α-L-fucosidase in white blood cells was 0.08 nmol/h.mg (normal range 31.44–104.84). The patient was eventually diagnosed with fucosidosis.

## Discussion and conclusions

Fucosidosis is an extremely rare disease. To our knowledge, this is the seventh case reported from China [2, 6]. Its clinical features were similar to those of some LSDs, such as MPS and ML. According to the literature, about 95% showed intellectual disability, 87% presented with movement disorders, 78% had coarse facial features, 78% had recurrent respiratory infections, 78% had growth retardation, 58% had dysostosis multiplex, 52% had diffuse telangiectasia or angiokeratoma on the skin which showed an obvious increase in incidence with age, 44% showed liver and spleen enlargement, and 38% had epilepsy [7]. Other clinical symptoms, including inguinal hernia, dystonia, visual impairment and corneal opacity, were also reported [1]. Our patient presented with intellectual disability, movement disorder, light coarse facial features, recurrent respiratory infections, growth retardation, dysostosis multiplex, diffuse angiokeratoma on the skin, hepatomegaly, dystonia, and inguinal hernia. These symptoms were consistent with clinical features in previous studies. For patients presenting with these above symptoms, fucosidosis should be considered except for MPS.

Imaging examination plays an important auxiliary role in the diagnosis. In the vast majority of case reports, the brain MRI shows symmetrical hyperintensity of the globus pallidus on T1-weighted images and symmetrical hypointensity of the globus pallidus with a linear high-intensity zone in the middle separating its medial and lateral segments on T2-weighted images. There are diffuse symmetric high signal zones in bilateral subcortical and deep white matter on T2-weighted images. Low signal of the thalami on T2-weighted is an important sign which prompts LSDs [8–11]. Early in the disease, cranial Computed Tomography (CT) shows increased cerebellar volume. In the late stages, cranial CT demonstrates diffuse atrophy both in cerebrum and cerebellum [1, 12]. In X-ray, patients of fucosidosis often present with dysostosis multiplex, such as anterior beaking of thoracolumbar vertebrae, lumbar hyperlordosis, kyphosis, scoliosis, small fifth lumbar vertebrae, short odontoid, widened and sclerotic long bone diaphysis, widened and scalloped acetabular roof. In addition, the skull will be thickened, and paranasal sinuses will be absent or hypoplastic. In summary, fucosidosis often show bone deformity and destruction in X-ray [12].

Fucosidosis should be suspected when patients present with the above clinical manifestations, imaging characteristics, and negative results of urinary GAGs. Moreover, enzyme activity detection suggesting low activity of α-L-fucosidase is the gold standard for diagnosis. Unfortunately, enzyme activity detection is not available in most hospitals in China. By comparison, high-throughput sequencing is more accessible in China and plays an important role in the diagnosis. Through the combined analysis of the clinical manifestations, typical MRI and X-ray findings, low activity of α-L-fucosidase and a homozygous frameshift variant of FUCA1 gene, our patient was finally diagnosed with fucosidosis at 7 years old.

Treatment means for this disease are given priority to symptomatic treatment currently. According to relevant literature, hematopoietic stem cell transplantation has been used in a few cases with the results of a significant increase in α-L-fucosidase activity and the improvement of partial clinical symptoms after earlier transplantation. But the long-term effects and complications of this treatment are undefined [13, 14]. The critical treatment for other LSDs like MPS is enzyme replacement therapy which is an undergoing preclinical study for fucosidosis [14, 15]. Gene therapy is also one of the key research directions in the future.

We report a case of fucosidosis which is caused by a homozygous pathogenic variant in the FUCA1 gene. Combined with enzyme activity detection and genetic testing, we got a definite diagnosis. In summary, when patients with similar clinical manifestations, brain MRI revealed symmetrical hypointensity of the globus pallidus with a linear high-intensity zone in the middle on T2-weighted images, and negative results of urinary GAGs, fucosidosis needs to be suspected. Genetic testing and/or enzyme activity detection help to make a definite diagnosis.

## Data Availability

The datasets used and/or analyzed during the current study are available from the corresponding author upon reasonable request.

## Abbreviations

LSDs: Lysosomal storage diseases
MPS: Mucopolysaccharidosis
ML: Mucolipidosis
GAGs: Glycosaminoglycans
MRI: Magnetic Resonance Imaging
CT: Computed Tomography
